# Factors Influencing the Settlement Intentions of Chinese Migrants in Cities: An Analysis of Air Quality and Higher Income Opportunity as Predictors

**DOI:** 10.3390/ijerph17207432

**Published:** 2020-10-13

**Authors:** Bo Li, Qingfeng Cao, Muhammad Mohiuddin

**Affiliations:** 1 School of Management, Tianjin University of Technology, Tianjin 300384, China; lb2088@email.tjut.edu.cn; 2 Institute of Modern Economic and Management, Tianjin University of Finance and Economics, Tianjin 300222, China; 3 Faculty of Business Administration, Laval University, Quebec, QC G1V 0A6, Canada

**Keywords:** air quality, income, settlement intention, migrants

## Abstract

With rapid urbanization, the air pollution issue is becoming an increasingly serious issue given that people are strongly swayed in their location choice to settle down in a growing urban area where most job opportunities have been created. This study investigated the influences of both air quality and income on the settlement intentions of Chinese migrants by using microlevel samples of the China Migrants Dynamic Survey (CMDS) data from 2017 and the annual average concentration of PM2.5 (particles with diameter ≤ 2.5 μm in the air) to measure a city’s air quality. The results showed that the settlement decisions of Chinese migrants involved a trade-off between income and air quality. Poorer air quality could significantly decrease the settlement intention, while a higher income could significantly increase the settlement intention of Chinese migrants. However, as the migrants’ income opportunity increased at a location, the negative influence of poorer air quality on the settlement intention at that location gradually declined. Specifically, when deciding whether to settle down in cities, the migrants with a non-agricultural “hukou” (household registration) tended to pay more attention to air quality than the migrants with an agricultural “hukou,” and migrants who moved farther away in geographic distance tended to pay more attention to income. It was concluded that the influences of air quality and income on the settlement intentions of the migrants were robust and consistent after using different estimation methods and considering the issue of endogeneity.

## 1. Background

Rapid industrialization and urbanization are changing the urban landscape in emerging markets [1]. One of the most profound changes affecting human society is the massive migration to cities to establish new livelihoods. This will eventually lead to the reconstruction of urban spaces and the integration of human lifestyles, both socially and economically. According to the International Migration Report 2017, there exist 253 million international migrants around the world, which have increased by approximately 49% since 2000, accounting for 3.4% of the total global population [2]. With the development of the global economy, most employees have gradually moved into regions with abundant economic activities and job opportunities. Furthermore, large cities offer lucrative attractions regarding the settlement intention of migrants because of the superior economic and non-economic incentives, including social, medical, educational, and municipal infrastructures that city dwellers enjoy [3].

In the past few decades, cities around the globe have experienced unprecedented growth, providing millions of people with social mobility and economic prosperity. However, the proliferation and continuous growth of urban areas have brought about many problems and challenges for the future of sustainable urban development [4]. One of the main consequences of urban expansion caused by rapid urbanization, population growth, and changes in consumption patterns is the gap between the rich and the poor in modern cities, the generation of large amounts of urban waste and urban pollution [5], and the increasingly serious environmental degradation [6]. Risk society theory emphasizes the importance of solving these ecological problems [7], which are new challenges facing the current rapid expansion of urban immigrants and urban life.

Therefore, migrants have contributed greatly to promoting global urbanization [8,9,10]. Especially in China, which is one of the most important advanced emerging economies, the accelerating transformation and upgrading of economic development and the rapidly growing agricultural productivity in recent decades have released millions of rural labor workers to pursue industrial activities in cities. Statistics show that in 2018, the number of Chinese migrants was 241 million, accounting for 17.3% of the total population [11]. Since the early 1980s, a large number of people have migrated from rural areas to cities and towns [12]. The emergence and growth of urban migrants have become inevitable phenomena in the process of Chinese industrial transformation and economic development, and have been one of the most important reasons for the rapid urbanization of China [13,14]. Therefore, issues related to urban migrants and the location choice as part of their settlement intentions become important. However, because of historical reasons, the differences in social and economic welfare between the migrants and residents in cities persist, especially in the “hukou” (household registration) system. The “hukou” system was established in 1958 to regulate the flow of rural migrants into cities [15,16]. The “hukou” system not only involves compulsory registration for the population but is also a formal and legal way to bind the related welfare of urban residents [17], which has been one of the key institutional factors in the settlement decisions for the migrants into cities [18].

Recent research on migrants’ location choices regarding settlement intentions in urban areas has investigated the demographic characteristics of migrants (such as age, marital status, educational level), family factors (such as family size), economic factors (such as income level), and social factors (such as the characteristics of employment and social security) [19,20,21,22,23]. In addition to the above factors, air pollution has become one of the most significant health risks faced by urban residents around the world and, to a certain degree, influences the settlement intentions of migrants. In 2017, United Nations Environment Programme (UNEP) found that the number of annual premature deaths was over 7 million worldwide due to air pollution. Therefore, the air quality, income, and cost of living in cities have significant influences on the perception of urban livability and the settlement decisions of migrants [24,25,26,27]. As the largest emerging economy in the process of rapid urbanization, China is faced with increasingly serious challenges regarding the air pollution in cities, which may pose a formidable challenge to the settlement intentions of migrants and the urbanization process. Therefore, exploring the influences of both urban air quality and income on the settlement intentions of migrants and investigating the characteristics and functional rule of their interactions are of great significance for promoting both the sustainable development of urbanization and the livability of cities in emerging markets, such as China.

An increasing number of studies have put emphasis on various factors that affect the settlement intentions of migrants in urban areas. From the macrolevel perspective, the economic, institutional, and cultural considerations have been emphasized as the key factors influencing the settlement intentions of migrations. Boccagni put forward the idea that the social and economic conditions of original residential places may accelerate the moving out of such migrants to more livable destinations [28]. Ette et al. pointed out that socio-cultural and institutional factors are among the decisive factors regarding the settlement intentions of migrants to another area, such as an urban area [29]. From the microlevel perspective, individual characteristics, human capital, integration, and connections with the home regions of migrants are the critical influencing factors regarding settlement intentions to another region. Paparusso and Ambrosetti found that microlevel factors in Italy, such as socio-economic and work conditions, determined the migration intentions of Moroccans [30]. Studies have been done on the influencing factors of the settlement intentions of Chinese migrants, focusing on economic, socio-cultural, institutional, individual, and family factors. Among the previous studies, the economic factors are the primary influence on location choice regarding settlement intentions, where the higher the expected or actual income, the higher the settlement intention of Chinese migrants into cities [27,31]. Liang proposed to improve the living conditions and increase the income of Chinese migrants, which would be helpful for the promotion of social integration into city life [32]. Different from the early generations of Chinese migrants, who strived to achieve an optimized level of economic status for both themselves and their families through working in cities far away from hometown and family [33], new Chinese migrants tend to take social and cultural factors into consideration when making their settlement decisions [34]. Abundant social capital, smooth social integration, and voluntary cultural adaptation are considered as positive factors that influence the sense of belonging and the settlement intentions of Chinese migrants [22].

In brief, the existing research has comprehensively studied both the macro- and micro-level influencing factors on the settlement intentions of migrants but the decisive roles played by ecological and environmental factors in the settlement intentions have rarely been addressed by researchers. According to the International Organization for Migration (IOM), “environmental immigration” refers to those who voluntarily leave or are forced to leave their residential places temporarily or permanently due to sudden or gradual environmental degradation [35]. The academic community is increasingly addressing the influences of environmental conditions on the settlement intentions of migrants. In fact, environmental livability plays an important role in migrants’ decisions regarding participating in the urban labor market, thus affecting their incomes and life satisfaction [36,37,38]. The study of Tiebout mainly focused on the influences of non-economic factors, such as the urban living environment quality on the settlement intentions of migrants [39]. Similarly, other researchers have also confirmed the positive influences of urban life quality [40,41] and comfortability [42] on the settlement intentions of migrants. However, many types of environmental problems, such as gradual environmental degradation, soil degradation, declining vegetation, and global warming, may also affect the settlement intentions of migrants [43,44]. Especially in China, with the increasing demands for a more livable environment from urban residents, urban environment-related factors, such as living conditions [45] and environment quality [46], are found to have greater influences on the settlement intentions of migrants into Chinese cities.

In brief, it is necessary to further investigate the influences of environmental factors on the decisions of migrants, especially the air quality and its interaction with the individual incomes of migrants.

## 2. Model and Data

In general, this study used the China Migrants Dynamic Survey (CMDS) data from 2017 to measure the urban air quality in terms of the annual average concentration of PM2.5 (particles with diameter ≤ 2.5 μm in the air) and design regression models. Furthermore, this study used the monthly salaries and net incomes of respondents in the CMDS 2017 as the indicator for the incomes of Chinese migrants into cities and conducted an empirical study of the influences on the settlement intentions.

### 2.1. Chinese Migrants’ Data

The dataset of the Chinese migrants used in this study came from the latest CMDS, published in 2017, which was released by the Migrant Population Service Center of the National Health Commission of China (http://www.chinaldrk.org.cn). The CMDS is the most detailed microlevel survey data about Chinese migrants. The survey respondents are the residents who are 15 years old and above that are not registered in the district (county, city) and have resided in their immigratory city for more than one month. The survey covers 31 provincial areas in China, including autonomous regions and municipalities. In the questionnaire of the CMDS 2017, respondents were required to answer questions such as “If you plan to stay here, how long do you plan to stay?”. The six alternative answers for this question were “1–2 years,” “3–5 years,” “6–10 years,” “more than 10 years,” “settle down,” and “not sure.” This question was used to identify those who have settlement intentions. Specifically, respondents who answered “settle down” could be considered to have a settlement intention (*SI*), where the value of variable *SI* was set to 1; otherwise, 0. Meanwhile, this study used the monthly salaries or net incomes of respondents in the dataset from the CMDS 2017 to represent the incomes of Chinese migrants in the sample, which excluded missing values and the minimum 1% (income <200 yuan) and the maximum 1% (income >20 thousand yuan) outliers to finally obtain 123,338 observations.

Specifically, migrants who had a settlement intention accounted for 43.20% of the migrants with a non-agricultural “hukou,” while migrants who had a settlement intention only accounted for 23.67% of the migrants with an agricultural “hukou.” Therefore, migrants with a non-agricultural “hukou” had a higher ratio of settlement intention. The reason for this was that under the current Chinese “hukou” system, there still exist certain institutional constraints regarding the settlement of migrants with an agricultural “hukou” in cities. These constraints in turn lead to the lower settlement intentions of Chinese migrants with an agricultural “hukou.” Meanwhile, most migrants with a non-agricultural “hukou” in China are peasant workers [12,47]. Their income cannot support their lives in the cities. Hence, they often choose to work in cities but settle down in rural areas [48].

Table 1 reports the ratios of migrants with different levels of education who had settlement intentions. It can be found that the group of migrants with the highest proportion having settlement intentions (58.84%) was the population with a graduate level of education. However, the group of migrants with a proportion having settlement intentions lower than 20% was the population with a junior middle school level of education or less. In other words, the higher the education levels of the migrants in the observations, the stronger their settlement intentions.

### 2.2. PM2.5 Data of Chinese Cities

In recent years, with the acceleration of the Chinese urbanization process, PM2.5 has become one of the most important factors affecting the air quality and has caused frequent air pollution events in Chinese cities [49]. In recent years, hazy weather caused by multiple pollutants, especially represented by PM2.5 as the main pollutants, has affected large areas of China, lasting for a long time [50]. With the rapid economic development, China is suffering from serious air pollution, where PM2.5 has gradually become the primary pollutant, which has attracted widespread social concern [51,52]. The existing studies also show that PM2.5 is an important factor affecting China’s population mobility. In China, with the development of society and the improvement of living conditions, people’s demands on the living environment are gradually increasing.

Furthermore, interregional migration in China is no longer only determined by the levels of regional economic development and social employment. Having a favorable environment in any given region also significantly influences the location decisions of Chinese migrants, which can provide sustainable human capital for economic development and increase the external benefits of a favorable environment in the region [53]. Therefore, population mobility not only depends on the quality of the economic conditions but also on the living environment, which has become an increasingly important indicator for people to consider. In particular, air quality is gradually becoming an important indicator for people to judge the quality of a living environment [54].

The PM2.5 concentration can be calculated using satellite remote sensing data, which exhibits higher accuracy than air quality data from other sources. Therefore, this study used the annual average concentration of PM2.5 (μg/m^3^) to measure the air quality in Chinese cities. Due to the inaccessibility of the 2017 PM2.5 dataset of Chinese cities from official channels, this study used the satellite-based grid data on the global PM2.5 concentrations released by the Atmospheric Composition Analysis Group at Dalhousie University and used ArcGIS 10.2 (Esri, Redlands, CA, USA) to calculate the annual data of the average PM2.5 concentrations of Chinese cities in the prefecture-level cities or above in 2017. The results showed that the average annual PM2.5 concentration in 2017 in the sample of Chinese cities was 44.80 μg/m^3^. Among the results, the highest annual average concentration of PM2.5 was 80.66 μg/m^3^ in Hengshui city of Hebei province and the lowest annual average concentration of PM2.5 was 10.02 μg/m^3^ in Hulunbuir city of Inner Mongolia.

The spatial distribution of the PM2.5 concentration in each Chinese city in 2017 in the sample exhibited significant differences. A city’s PM2.5 concentration was highly correlated to its economic development level, industrial structure, and natural environment [55]. For example, almost all the cities in the developed provinces of China, including Shandong, Jiangsu, Zhejiang, Shanghai, and Beijing, had higher PM2.5 concentrations. Simultaneously, cities in provinces with higher ratios of secondary industries, including Hebei and Tianjin, also had higher PM2.5 concentrations. However, because of the humid and rainy climate, the PM2.5 concentrations in the cities of southern China were not so high.

### 2.3. Regression Model

According to the seminal work of Henderson [56] and Roback [57], when a spatial equilibrium is achieved, the levels of residents’ utilities in different regions are the same. Since migrants always tend to move to the cities with higher levels of utility, whether the migrants choose to settle down or not depends on the utility levels in cities. In addition to income, which is an important influencing factor on the utility levels of migrants, satisfactory air quality can also improve their subjective wellbeing [58,59]. Therefore, the higher the income and air quality of cities, the higher the levels of utility experienced by the migrants, and hence the higher the probability of settling down in those cities, with everything else being equal [60]. However, in many cases, high income and satisfactory air quality are incompatible. The existence of an environmental Kuznets curve means that the relationship between income and air quality tends to be an inverted U shape [61,62,63]. Especially in emerging countries, such as China, which is a developing country with relatively low per capita income, areas with a higher income often face more serious air pollution. Therefore, whether migrants choose to settle down in a city or not is a trade-off between higher income and poorer air quality. Although poorer air quality can significantly decrease the settlement intentions of migrants, a higher income will often compensate for the resulting loss of utility of the migrants. In order to test the influences of both air quality and income on the settlement intentions of the migrants, a regression model was built, as follows:

(1) $$SI_{ij}=\beta_{0}+\beta_{1}PM2.5_{ij}+\beta_{3}income_{ij}+\lambda X+\rho Z+\mu_{ij}$$

(2) $$SI_{ij}=\beta_{0}+\beta_{1}PM2.5_{ij}+\beta_{2}PM2.5_{ij}\times income_{ij}+\beta_{3}income_{ij}+\lambda X+\rho Z+\mu_{ij}$$

where *i* represents the individual, and *j* represents the Chinese city. *SI_ij_* stands for settlement intention of the migrant, *PM2.5_ij_* represents air quality of the cities, and *income_ij_* represents the monthly income of migrant *i* in city *j*. Since the explained variable *SI* of model (1) is a binary dummy variable, this study used a logistic regression model to estimate model (1). Note that when estimating a binary choice model, such as model (1), a logistic regression model and a probit regression model are equivalent [64]. This study also estimated model (1) using a probit regression model in the following robustness test. A logistic regression model is a nonlinear model, where the coefficients of variables in model (1) are not the marginal effects, as in a linear regression model, but their signs are consistent with the marginal effects [64]. By substituting the estimated coefficients in model (1) into the exponential function with log-base e, the odds ratio was obtained. According to the theoretical analyses above, *β_1_* < 0, *β_2_* > 0, and *β_3_* > 0 was expected and assumed.

Furthermore, *X* represents a vector that included all the control variables of individual characteristics that affect the settlement intention of the migrant (*nation*, *gender*, *age*, *party*, *edu*, *hukou*, *marriage*, *time*, *distance^1^*, *distance^2^*, *distance^3^*, *reason*). All control variables came from the CMDS 2017. *Z* represents a vector including other city-level variables that affected the settlement intentions of migrants (*third*, *trade*, *pgdp*, *gdpr*), which all came from the China City Statistical Yearbook 2018. In addition, the provincial fixed effect in model (1) was also controlled, and $\mu_{ij}$ represents the residual term. Model (2) added an interaction term *PM2.5 ×* *income* based on model (1).

The specific definitions and descriptive statistics for all variables are reported in Table 2 and Table 3.

## 3. Results and Discussion

### 3.1. Basic Results

Table 4 reports the estimated results of model (1) and model (2). Among them, column (1) exhibits the results from model (1), while column (2) represents the results from model (2). Specifically, the coefficient of the variable *PM2.5* in column (1) of Table 4 was significantly negative at the level of 5%, which indicated that with all else being equal, an increase in the PM2.5 concentration could decrease the settlement intentions of Chinese migrants. In other words, when increasing the annual average PM2.5 concentration of Chinese cities by 1 μg/m^3^, the odds ratio of the settlement intentions of Chinese migrants decreased by 0.30% (calculated using 1 − e^−0.003^). Simultaneously, the coefficient of the variable *income* was significantly positive at the level of 1%, which showed that the higher the incomes that the migrants earned from the city, the stronger the settlement intentions of the migrants. That is, with all else being equal, the odds ratio of the migrants to settle down in the city increased by 12.76% when the monthly incomes of the migrants increased by 10,000 yuan (calculated using 1 − e^−0.755^). Because poorer air quality can decrease the utility levels of the migrants, and higher incomes can increase the utility levels of the migrants, the signs of the coefficients of the above two variables were consistent with the theoretical expectations.

**Figure 1:**
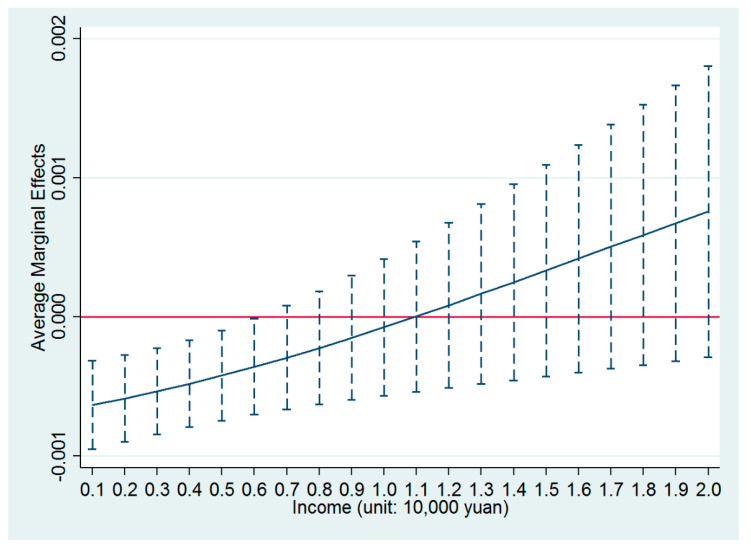
Average marginal effects of PM2.5 with 90% confidence intervals.

After including the interaction term *PM2.5 ×* *income,* the regression result of column (2) in Table 4 shows that the coefficient of *PM2.5 ×* *income* was significantly negative, which confirmed the trade-off between a higher income and poorer air quality that was faced by the migrants when deciding whether to settle down in a city. In other words, the influence of air quality on the settlement intentions of Chinese migrants was conditional on their incomes. As income opportunities increased at a migrant’s location, the negative influence of poorer air quality on the settlement intention at that location gradually decreased, which indicated that a higher income could make up for the lost utility caused by poorer air quality. Figure 1 further shows the average marginal effect of the variable *PM2.5* on the settlement intentions of Chinese migrants. As shown in the figure, with the increasing income of the migrants, the average marginal effect of PM2.5 gradually increased from significantly negative to eventually being insignificant. Therefore, the results above indicated that a higher income weakened the negative influence of poor air quality on the settlement intentions of Chinese migrants.

According to the results of the coefficients of the individual control variables, migrants with the following properties had stronger settlement intentions: non-Han ethnic group, female, married, CCP member or CCYL member, higher educational level, a non-agricultural “hukou,” a longer duration of residence in migratory cities, a shorter migration distance, and a non-economic purpose. With the increase of age, the settlement intentions of Chinese migrants first increased and then decreased, which was basically consistent with the existing studies [65,66,67].

According to the results of the city-level control variables, Chinese migrants were more willing to settle down in the cities with higher proportions of tertiary industries that accommodate the major part of the employed migrants. In our samples, 59.3% of the migrants were employed in a tertiary industry. Meanwhile, the settlement intentions of the migrants were stronger in the cities with a higher level of openness to international trade, as international trade creates a lot of employment opportunities [68]. In addition, Chinese migrants tended to settle down in the cities with a higher per capita GDP (not significant) and a higher GDP growth rate.

### 3.2. Heterogeneity Test

**Figure 2:**
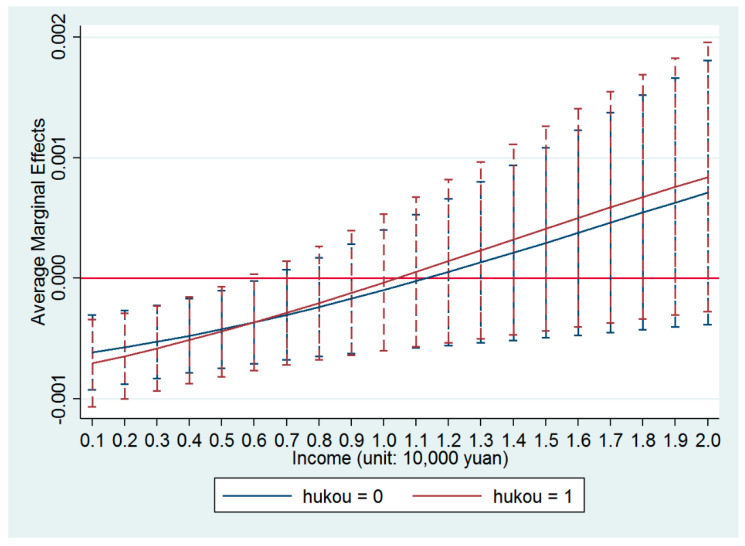
Average marginal effects of PM2.5 with 90% confidence intervals for the migrants of different “hukous.”

The heterogeneity in the influence of both air quality and income on the settlement intentions of Chinese migrants was mainly tested in terms of the following two aspects:

(1) Heterogeneity of the migrants’ “hukou.” Under the current “hukou” system in China, the “hukou” of the migrants can be divided into two types: non-agricultural “hukou” and agricultural “hukou.” The migrants with a non-agricultural “hukou” are mainly city residents, while the migrants with an agricultural “hukou” are mainly rural workers who moved from rural areas to cities. The preferences of the migrants of these two types in terms of both income and air quality may be significantly different. Based on model (2), the following model could be further estimated:

(3) $$\;SI_{ij}=\alpha_{0}+\alpha_{1}PM2.5_{ij}+\underset{h=0,1}{\sum }\alpha_{h}PM2.5_{ij}\times income_{ij}\times hukou_{ij}^{h}+\lambda X+\rho Z+\mu_{it}$$

where the coefficients *α_h_* (*h* = 0 or 1) of the interaction term *PM2.5 × income × hukou^h^* represents the difference in the preferences of income and air quality in the settlement decisions of Chinese migrants with either an agricultural “hukou” or a non-agricultural “hukou.” Column (1) in Table 5 reports the estimated results of the coefficients of the variables in model (3), which showed that the coefficients of the interaction term *PM2.5 × income × hukou^h^* were significantly positive. On this basis, Figure 2 reports the average marginal effects of PM2.5 on the settlement intentions of the migrants with different types of “hukou.”

It was found that the negative influences of PM2.5 on the settlement intentions of Chinese migrants gradually became insignificant with the increase of their incomes, no matter whether they had an agricultural “hukou” or a non-agricultural “hukou,” which was consistent with the conclusion drawn from Figure 1. However, with the increasing incomes of Chinese migrants, the negative influence of PM2.5 on the settlement intentions of Chinese migrants with a non-agricultural “hukou” decreased at a faster speed, which indicated that the migrants with a non-agricultural “hukou” paid more attention to the air quality than those with an agricultural “hukou” when deciding whether to settle down at a location in the city. In other words, in order to get higher incomes, Chinese migrants with an agricultural “hukou” were more tolerant of poorer air quality than those with a non-agricultural “hukou.” The main reason for this was that rural workers with an agricultural “hukou” tended to have lower environmental awareness [69] and paid more attention to incomes in the settlement decision. Gu and Ma [46] also found that Chinese migrants show an indifferent attitude toward the environmental problems in their immigratory cities in their case study of Shenzhen, which is one of the most developed cities in China.

**Figure 3:**
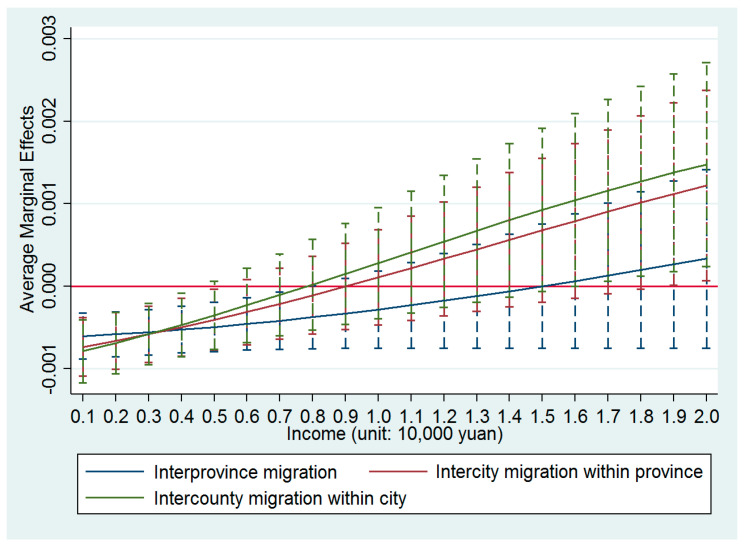
Average marginal effects of PM2.5 with 90% confidence intervals for migrants migrating over different migration distances.

(2) Heterogeneity of the migrants’ migration distance. According to the migration distances, the sample was divided into three subsamples of intercounty migration (within the same city), intercity migration (within the same province), and interprovince migration. In order to test the heterogeneous effects of both income and air quality on the settlement intentions of the migrants with different migration distances, the following model based on model (2) was estimated:

(4) $$SI_{ij}=\gamma_{0}+\gamma_{1}PM2.5_{ij}+\overset{3}{\underset{d=1}{\sum }}\gamma_{d}PM2.5_{ij}\times income_{ij}\times distance_{ij}^{d}+\lambda X+\rho Z+\mu_{it}$$

where the coefficient *γ_d_* (*d* = 1, 2, 3) of the interaction term *PM2.5* *× income* *× distance^d^* represents the preferences for income and air quality in the settlement decision of migrants with different migration distances. Column (2) in Table 5 reports the estimated results of model (4), which shows that the coefficient of the interaction term *PM2.5* *× income* *× distance^d^* was significantly positive. The average marginal effects of PM2.5 for migrants with different types of migration distances are shown in Figure 3.

According to Figure 3, with the increase of income, the negative influence of PM2.5 on the migrants’ settlement intentions decreased the fastest in the intercounty (within the same city) samples, followed by the intercity (within the same province) samples, and finally the interprovince samples. That is to say, migrants with a longer migration distance preferred income and were less likely to substitute income for air quality in their settlement decisions. The main reason for this was that 92.73% of Chinese migrants in the sample made their migration decisions for economic reasons, such as working or doing business, to improve their incomes. For all migrants with economic reasons for migrating, the migrants that undertook interprovince migration, intercity migration, and intercounty migration accounted for 50.14%, 32.83%, and 17.03%, respectively. Hence, migrants with a longer migration distance paid more attention to income than air quality.

### 3.3. Robustness Test

**Figure 4:**
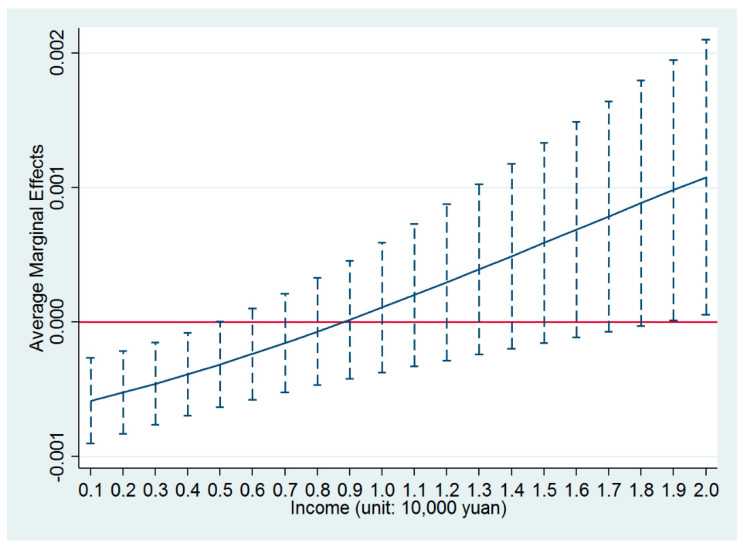
Average marginal effects of PM2.5 with 90% confidence intervals using the probit regression model.

This study used different estimation methods. In particular, the logistic regression model was used to estimate model (2), the results of which are reported in the previous tables. Next, this study further used the probit regression model to re-estimate the model. Column (1) in Table 6 reports the estimated results. It was found that the coefficient of the variable *PM2.5* was significantly negative at the level of 1%, the coefficient of the variable *income* was significantly positive at the level of 1%, and the coefficient of the interaction term *PM2.5 ×* *income* was significantly positive at the level of 1%. As for the average marginal effect of the variable *PM2.5*, it is clearly shown in Figure 4 that the negative influences of PM2.5 on the settlement intentions of the migrants gradually became insignificant with the increase of income, which was consistent with the results presented in Table 4.

Next, this study controlled for the endogeneity in the variable *PM2.5*. The baseline regression used in this study was the microlevel cross-sectional data model. However, the variable *PM2.5* was a variable at the city level. The PM2.5 concentration of a city may be correlated with other unobservable factors affecting migrants’ settlement intentions in the residuals *μ_ij_*, thus causing potential endogeneity problems. In order to control for this potential endogeneity, instrumental variables were used to re-estimate the probit regression model. In terms of the selection of the instrumental variables, this study calculated the average PM2.5 concentrations $\;\bar{PM2.5}$ of each city during 2015–2016 as the instrumental variable of *PM2.5*, and used $\bar{PM2.5}\times income$ as the instrumental variable of *PM2.5 × income* accordingly. The PM2.5 concentration of a city tends to be relatively stable in the short term, where the correlation coefficient of *PM2.5* and $\bar{PM2.5}$ in the sample was 0.96, and thus the instrumental variables were highly correlated with the endogenous variables. Furthermore, since $\bar{PM2.5}$ was a lagging variable, the correlation of $\bar{PM2.5}$ and the city level unobservable factors in the residual term $\mu_{ij}$ could be theoretically excluded; therefore, $\bar{PM2.5}$ satisfied the condition for an instrumental variable. The column (2) in Table 6 shows the estimation results using the instrumental variables, where it was found that the results were basically consistent with the results of column (1) in Table 6. Therefore, the robustness of the relationship between the influences of air quality and income on migrants’ settlement intentions was confirmed.

### 3.4. Discussion

With the gradual progress of the Chinese urbanization process, it is crucial to advance the smooth integration of the migrants into cities to further achieve sustainable development in terms of both modernization and urbanization. For emerging countries with a relatively low urbanization ratio, improving the living environment and income level of the migrants can increase their settlement intentions and integration into city lives. The empirical research based on the Chinese dataset used in this study indicated that the migrants often faced a trade-off between income and air quality in their settlement intentions. In varying degrees, the migrants tended to tolerate poorer air quality to obtain higher incomes. In order to break through this dilemma, it is necessary to find a way out of the traditional development pattern indicated by the left side of the environmental Kuznets curve, in which economic growth tends to aggravate environmental pollution.

On the one hand, green and environmentally friendly industries should be encouraged in the cities of emerging industrializing countries. The coordinated development model of both economic growth and environmental protection needs to be further explored. At the same time, the government should strengthen environmental regulations, reinforce the prevention and control of environmental pollution, and keep engaging in the continuous improvement of the environment during economic development. Furthermore, it is of great importance to take environmental governance as one of the most important goals of urban development to further improve the livability of cities for both local residents and migrants.

On the other hand, regional coordinated development models should be established to reduce the gaps in both income inequality and environmental awareness between Chinese cities. At present, there exist significant differences in economic development and the level of the livability of cities in China. Therefore, migrants tend to settle down in the cities with lower air quality but higher incomes. Therefore, it is necessary to further promote coordinated economic development between cities, innovate the existing household registration system, and use the development of urban agglomerations or city clusters to drive economic development and employment in the small- and medium-sized cities surrounding metropolitans. Furthermore, the economic development and awareness of environmental health in less developed regions of emerging countries, such as China, should be promoted and encouraged. It is critical to promote the optimization of the regional industrial layout and spatial agglomeration and nurture the environmental awareness of residents to achieve an optimized balance between economic development and environmental health in emerging countries.

## 4. Conclusions

Using a microlevel sample from the China Migrants Dynamic Survey data from 2017 and the annual average concentration of PM2.5 to measure city air quality, this study investigated the influences of both air quality and income on the settlement intentions of Chinese migrants. The results of this study showed that when making a settlement decision, Chinese migrants were faced with a trade-off between poorer air quality and higher income. Poorer air quality could significantly decrease the settlement intentions of the migrants, while a higher income could significantly increase the settlement intentions. However, the negative influences of poorer air quality on the settlement intentions of the migrants gradually decreased with the increasing income opportunities of the migrants at that location. This seems to be a bit of an unreasonable choice that was made by the migrants, as generally, people tend to live in an environmentally safer place with the rise of income. The findings implied that the Chinese rural migrants moving into cities were still financially weak and sacrificed their wellbeing for higher income opportunities. They had not yet crossed the income threshold from where they would prioritize settling down in an environmentally safer place over a location with a higher income opportunity but was environmentally less safe.

Furthermore, there existed an apparent heterogeneity in the influences of both air quality and income on the settlement intentions of the migrants with different “hukous” and migration distances. Specifically, when deciding whether to settle down in a city, Chinese migrants with a non-agricultural “hukou” paid more attention to air quality than the migrants with an agricultural “hukou,” while the migrants with an agricultural “hukou” were more tolerant of poorer air quality than migrants with a non-agricultural “hukou.” Furthermore, the longer the migration distance of the Chinese migrants, the more emphasis that was put on the income when making settlement decisions. In addition, this study also used different estimation methods in the robustness test and controlled for potential endogeneity using instrumental variables. The robustness of the relationship between air quality and income regarding their influences on the settlement intentions of the migrants was confirmed.

## Figures and Tables

**Table 1:** The proportions of migrants with different levels of education who had settlement intentions.

Level of Education	Proportion
Never gone to school	19.22%
Primary school	17.82%
Junior high school	21.31%
Senior high school	30.51%
Junior college	44.27%
Undergraduate	52.71%
Graduate or above	58.84%

**Table 2:** Definitions of variables.

Variable Name	Variable Definitions
*SI*	Binary dummy variable, standing for settlement intention of the migrant. If migrant *i* decides to settle down in city *j*, it is 1; otherwise, 0.
*PM2.5*	Air quality of cities, measured using the annual average PM2.5 concentration (μg/m^3^) of the Chinese city where the migrant resides.
*income*	Monthly personal income in the immigratory city of the migrant (10,000 yuan)
*nation*	Dummy variable, the national/ethnic group of the migrant: ethnic Han = 1, others = 0.
*gender*	Dummy variable, gender of the migrant: male = 1, female = 0.
*age*	Age of the migrant (years).
*marriage*	Dummy variable, marital status of the migrant: married = 1, unmarried = 0.
*party*	Dummy variable, political identity of the migrant: China Communist Party (CCP) member or Chinese Communist Youth League (CCYL) member = 1, otherwise = 0.
*edu*	Level of education, measured in terms of the educated years of the migrant: never gone to school = 0 year, primary school = 6 years, junior high school = 9 years, senior high school = 12 years, junior college = 15 years, undergraduate = 16 years, graduate or above = 19 years.
*hukou*	Dummy variable, the “hukou” status of the migrant: non-agricultural “hukou” = 1, agricultural “hukou” = 0.
*time*	Duration of residence in the immigratory city of the migrant (year).
*distance ^1^*	Dummy variable, spatial distance of migration for the migrant: migration crossing provincial border = 1, others = 0.
*distance ^2^*	Dummy variable, spatial distance of migration for the migrant: migration crossing city border = 1, others = 0.
*distance ^3^*	Dummy variable, spatial distance of migration for the migrant: migration crossing county border = 1, others = 0.
*reason*	Dummy variable, reason for migration for the migrant: economic purpose (work or business) = 1, non-economic purpose (trailing family member, marriage, or other reasons) = 0.
*third*	The proportion of tertiary industries in the city where the migrant resides in (%), expressed as the ratio between output value of the tertiary industry and the GDP.
*trade*	Dependence degree on the trade of the city where the migrant resides in (%), expressed as the ratio of the total export and import volumes to the GDP.
*pgdp*	Per capita GDP of the city where the migrant resides in (10,000 yuan).
*gdpr*	GDP growth rate of the city where the migrant resides in (10,000 yuan).

**Table 3:** Descriptive statistics of the variables.

Variable	Observations	Mean	Standard Deviation	Minimum	Maximum
*SI*	123,338	0.279	0.449	0.000	1.000
*PM2.5*	123,338	45.802	14.208	10.016	80.657
*income*	123,338	0.424	0.280	0.020	2.000
*nation*	123,338	0.928	0.258	0.000	1.000
*gender*	123,338	0.568	0.495	0.000	1.000
*age*	123,338	36.801	9.765	16.000	85.000
*marriage*	123,338	0.804	0.397	0.000	1.000
*party*	123,338	0.109	0.311	0.000	1.000
*edu*	123,338	10.385	3.272	0.000	19.000
*Hukou*	123,338	0.219	0.414	0.000	1.000
*time*	123,338	7.164	5.895	1.000	58.000
*distance ^1^*	123,338	0.495	0.500	0.000	1.000
*distance ^2^*	123,338	0.330	0.470	0.000	1.000
*distance ^3^*	123,338	0.174	0.380	0.000	1.000
*reason*	123,338	0.927	0.260	0.000	1.000
*third*	123,338	52.384	11.507	1.907	80.603
*trade*	123,338	34.520	37.228	0.055	161.271
*pgdp*	123,338	6.140	2.716	0.988	13.110
*gdpr*	123,338	7.476	1.732	−2.800	12.300

**Table 4:** Results of baseline regression for factors influencing the settlement intentions of Chinese migrants in cities.

Variables	Model (1)	Model (2)
*PM2.5*	−0.003 **	−0.005 ***
	(0.001)	(0.001)
*PM2.5 × income*		0.004 **
		(0.002)
*income*	0.755 ***	0.561 ***
	(0.027)	(0.090)
*nation*	−0.103 ***	−0.102 ***
	(0.029)	(0.029)
*gender*	−0.238 ***	−0.238 ***
	(0.015)	(0.015)
*age*	0.013 **	0.013 **
	(0.006)	(0.006)
*age ^2^*	−0.000 **	−0.000 **
	(0.000)	(0.000)
*marriage*	0.593 ***	0.593 ***
	(0.024)	(0.024)
*party*	0.120 ***	0.120 ***
	(0.024)	(0.024)
*edu*	0.139 ***	0.138 ***
	(0.003)	(0.003)
*Hukou*	0.390 ***	0.390 ***
	(0.018)	(0.018)
*time*	0.064 ***	0.064 ***
	(0.001)	(0.001)
*distance ^2^*	0.592 ***	0.591 ***
	(0.019)	(0.019)
*distance ^3^*	0.873 ***	0.873 ***
	(0.024)	(0.024)
*reason*	−0.800 ***	−0.799 ***
	(0.026)	(0.026)
*third*	0.011 ***	0.011 ***
	(0.001)	(0.001)
*trade*	0.001 ***	0.001 ***
	(0.000)	(0.000)
*pgdp*	0.003	0.003
	(0.005)	(0.005)
*gdpr*	0.041 ***	0.041 ***
	(0.008)	(0.008)
Constant	−4.549 ***	−4.477 ***
	(0.158)	(0.161)
Province Fixed Effects	Yes	Yes
Observations	123,338	123,338
Pseudo R^2^	0.149	0.149

Note: The robust standard errors are given in parentheses, **, and *** indicate statistical significance at the 10%, 5%, and 1% levels, respectively. The abbreviations are defined in Table 2.

**Table 5:** Heterogeneity test for factors influencing the settlement intentions of Chinese migrants in cities with interaction terms included.

Variables	(1)	(2)
*PM2.5*	−0.004 ***	−0.005 ***
	(0.001)	(0.001)
*PM2.5 × income × hukou ^0^*	0.0040 **	
	(0.002)	
*PM2.5 × income × hukou ^1^*	0.0043 **	
	(0.002)	
*PM2.5 × income × distance ^1^*		0.003 *
		(0.002)
*PM2.5 × income × distance ^2^*		0.005 ***
		(0.002)
*PM2.5 × income × distance ^3^*		0.006 ***
		(0.002)
*income*	0.563 ***	0.552 ***
	(0.090)	(0.090)
*nation*	−0.102 ***	−0.104 ***
	(0.029)	(0.029)
*gender*	−0.238 ***	−0.240 ***
	(0.015)	(0.015)
*age*	0.013 **	0.013 **
	(0.006)	(0.006)
*age ^2^*	−0.000 **	−0.000 **
	(0.000)	(0.000)
*marriage*	0.593 ***	0.592 ***
	(0.024)	(0.024)
*party*	0.120 ***	0.120 ***
	(0.024)	(0.024)
*edu*	0.138 ***	0.139 ***
	(0.003)	(0.003)
*hukou*	0.383 ***	0.392 ***
	(0.028)	(0.018)
*time*	0.064 ***	0.064 ***
	(0.001)	(0.001)
*distance ^2^*	0.591 ***	0.549 ***
	(0.019)	(0.028)
*distance ^3^*	0.873 ***	0.819 ***
	(0.024)	(0.035)
*reason*	−0.799 ***	−0.799 ***
	(0.026)	(0.026)
*third*	0.011 ***	0.011 ***
	(0.001)	(0.001)
*trade*	0.001 ***	0.001 ***
	(0.000)	(0.000)
*pgdp*	0.003	0.003
	(0.005)	(0.005)
*gdpr*	0.041 ***	0.041 ***
	(0.008)	(0.008)
Constant	−4.475 ***	−4.420 ***
	(0.161)	(0.163)
Province Fixed Effects	Yes	Yes
Observations	123,338	123,338
Pseudo R^2^	0.149	0.149

Note: The robust standard errors are given in parentheses. *, **, and *** indicate statistical significance at the 10%, 5%, and 1% levels, respectively. The abbreviations are defined in Table 2.

**Table 6:** Robustness test for the factors influencing the settlement intentions of Chinese migrants in cities using a probit regression model and the instrumental variables.

Variables	(1)	(2)
*PM2.5*	−0.002 ***	−0.005 ***
	(0.001)	(0.001)
*PM2.5 × income*	0.003 ***	0.002 **
	(0.001)	(0.001)
*income*	0.320 ***	0.357 ***
	(0.053)	(0.054)
*nation*	−0.058 ***	−0.061 ***
	(0.017)	(0.017)
*gender*	−0.140 ***	−0.141 ***
	(0.009)	(0.009)
*age*	0.008 **	0.008 **
	(0.003)	(0.003)
*age ^2^*	−0.000 **	−0.000 **
	(0.000)	(0.000)
*marriage*	0.331 ***	0.336 ***
	(0.014)	(0.014)
*party*	0.074 ***	0.076 ***
	(0.014)	(0.014)
*edu*	0.080 ***	0.080 ***
	(0.002)	(0.002)
*hukou*	0.238 ***	0.230 ***
	(0.011)	(0.011)
*time*	0.037 ***	0.037 ***
	(0.001)	(0.001)
*distance ^2^*	0.343 ***	0.339 ***
	(0.011)	(0.011)
*distance ^3^*	0.510 ***	0.502 ***
	(0.014)	(0.014)
*reason*	−0.478 ***	−0.477 ***
	(0.016)	(0.016)
*third*	0.006 ***	0.006 ***
	(0.001)	(0.001)
*trade*	0.001 ***	0.001 ***
	(0.000)	(0.000)
*pgdp*	0.002	0.002
	(0.003)	(0.003)
*gdpr*	0.023 ***	0.026 ***
	(0.005)	(0.005)
Constant	−2.600 ***	−2.487 ***
	(0.093)	(0.094)
Province Fixed Effects	Yes	Yes
Observations	123,338	123,338
Pseudo R^2^	0.149	0.148

Note: The robust standard errors are given in parentheses. **, and *** indicate statistical significance at the 10%, 5%, and 1% levels, respectively. The abbreviations are defined in Table 2.
